# Establishing a rapid-response multidisciplinary service for thrombotic microangiopathies using a model of improvement framework: perspectives from a Brazilian quaternary hospital

**DOI:** 10.3389/fmed.2026.1944769

**Published:** 2026-09-25

**Authors:** Rosana Moreira Cosentino Penteado, Adriana Cristina D'Ornelas, Nelson Martins Ferreira Junior, Carla Souza Behr Pitoli, Claudia Garcia de Barros, Sebastian Vernal, Ederson Haroldo Pereira de Almeida, Joao Carlos de Campos Guerra

**Affiliations:** 1 Departamento de Patologia Clínica, Medicina Diagnóstica, Hospital Israelita Albert Einstein, São Paulo, SP, Brazil; 2 Escritório de Excelência em Saúde, Hospital Israelita Albert Einstein, São Paulo, SP, Brazil; 3 Departamento de Oncologia e Hematologia, Hospital Israelita Albert Einstein, São Paulo, SP, Brazil

**Keywords:** electronic health records, hemathology, patient care team, quality improvement, thrombotic microangiopathies

## Abstract

Thrombotic microangiopathies (TMA) require prompt identification and coordinated treatment because they are associated with substantial morbidity, irreversible target-organ injury, and mortality. Yet fragmented diagnostic and care pathways often delay diagnosis and lead to poorer outcomes. This Perspective article describes the design and implementation of a TMA Center of Excellence at a Brazilian quaternary hospital. This initiative aimed to design and implement a rapid-response, multidisciplinary TMA service to shorten diagnostic and treatment delays, standardize diagnosis-specific care, and establish prospective measures to evaluate clinical and operational performance. Rather than reporting outcomes, we highlight design choices, stewardship safeguards for high-cost therapies, and practical implementation enablers. Multidisciplinary teams designed and tested diagnostic and therapeutic workflows using iterative Plan–Do–Study–Act cycles. Care pathways tailored to specific patient populations were embedded into the electronic medical record (EMR). EMR integration enabled real-time alerts triggered by key laboratory results, while embedded tools, such as the PLASMIC score (platelet count, hemolysis, no active cancer, no history of solid-organ or stem-cell transplant, mean corpuscular volume, international normalized ratio, and creatinine) and therapy-specific prescription menus, guided clinical decisions. A structured indicator framework was developed to prospectively monitor time to diagnosis, adherence to clinical pathways, length of stay, and intensive care utilization. Interdisciplinary collaboration facilitated alignment across emergency, radiology, laboratory, nephrology, and hematology services. This quality improvement strategy supports the feasibility of implementing a comprehensive, multidisciplinary model for TMA management and offers a transferable blueprint that middle-income health systems can adapt.

## Introduction

### The need for structured diagnosis and management of thrombotic microangiopathies

Thrombotic microangiopathies (TMAs) are a diverse group of serious disorders characterized by endothelial injury and platelet-rich microvascular thrombosis, leading to microangiopathic hemolytic anemia, thrombocytopenia, tissue ischemia, and potentially severe target-organ damage (1). They are typically associated with microangiopathic hemolytic anemia and thrombocytopenia (1).

Although they share a common feature—widespread damage to the endothelium and blood clot formation—TMAs include various clinical conditions such as thrombotic thrombocytopenic purpura (TTP), complement-mediated TMA (atypical hemolytic uremic syndrome or aHUS), and Shiga toxin-producing *Escherichia coli*-associated hemolytic uremic syndrome (STEC-HUS). Secondary TMA refers to TMA occurring in the context of an underlying disease or clinical condition, such as cancer, autoimmune diseases, pregnancy, infections, and certain medications (2–4); TTP is not included in this category. Transplant-associated TMA (TA-TMA) is considered separately as a distinct multisystem endothelial injury syndrome following hematopoietic cell transplantation, with complement activation contributing to its pathobiology (2–4).

The differential diagnosis of TMAs is both clinically and etiologically complex because several TMA syndromes and other conditions present with overlapping clinical and laboratory features, and clinicians often must make urgent treatment decisions (2). The multifactorial, often non-specific presentation of TMAs increases the risk of under-recognition, which may result in delayed diagnosis, progressive organ damage, and preventable deaths. Earlier diagnosis and collaborative input from specialists are therefore essential for better outcomes (5, 6). Accordingly, international guidelines advocate for multidisciplinary, protocol-based management as a key patient safety priority (5, 7–11).

The *Einstein Hospital Israelita* (EHI) in São Paulo, Brazil, approved a structured quality improvement (QI) project to establish a Center of Excellence for Diagnosis and Treatment of TMAs. The initiative launched a single-site pilot program incorporating scalable protocols, electronic alert pathways, dedicated multidisciplinary teams, and defined performance metrics. Its main goal is to reduce time-to-diagnosis, optimize therapy, and generate real-world evidence to inform potential broader implementation. This article describes the design and implementation of the initiative and discusses lessons that may inform similar efforts in other high-complexity settings in middle-income countries.

## Setting

The EHI operates a comprehensive healthcare system encompassing multiple facilities. The main hospital campus (Morumbi, São Paulo, Brazil), at the time of this initiative, comprises approximately 706 beds, including 37 adult medical-surgical intensive care unit (ICU) beds and 81 adult step-down unit beds. Additionally, the pediatric ICU consists of 15 beds.

## Conceptual framework for improvement

We conducted a hospital-based QI approach from February 2024 to April 2025. The project was integrated into the Institute for Healthcare Improvement (IHI) Model for Improvement (12) and used iterative Plan–Do–Study–Act (PDSA) cycles to test and refine changes on a small scale before hospital-wide implementation. The multidisciplinary team involved in the initiative consisted of internal and external specialists from various departments at EHI, including Obstetrics (2 specialists), Pediatrics (4 specialists), Intensive Care (4 specialists), Emergency (2 specialists), Transplantation (4 specialists), Pathology (1 specialist), Radiology (2 specialists), Dialysis (2 specialists), Laboratory (1 specialist), and Blood Bank (1 specialist).

### Intervention

To develop the institutional TMA response model, the multidisciplinary team conducted a thorough assessment of current clinical workflows to identify areas for improvement, potential system failures, and inefficiencies in patient care. This review informed subsequent multidisciplinary discussions held both virtually and in person. In a culture of shared learning—where everyone could teach and learn—the team built a comprehensive understanding of the TMA patient journey. This included detailed mapping of the diagnostic, treatment, and management steps for different patient groups: adults, children, pregnant women, kidney transplant recipients, and hematopoietic stem cell transplant recipients. From initial triage to diagnostic confirmation, each stage was carefully evaluated. As part of this process, the team developed and implemented clinical decision-support tools to standardize care pathways and encourage timely, evidence-based interventions.

The institutional pathways were informed by the 2020 International Society on Thrombosis and Haemostasis (ISTH) diagnostic (13) and treatment guidelines (14), the 2025 ISTH focused update (15), the 2023 British Society for Haematology guideline (16), and diagnosis-specific consensus statements (17, 18). For suspected immune TTP, these sources support early Caplacizumab alongside therapeutic plasma exchange and immunosuppression when clinically appropriate. Caplacizumab has been registered in Brazil by the Brazilian Health Regulatory Agency (ANVISA) (19) since 2021 for adults with acquired TTP, in combination with plasma exchange and immunosuppression; however, access may vary depending on formulary inclusion, reimbursement, and case-specific authorization.

### Implementation

After developing the clinical algorithms and care pathways, the multidisciplinary team established clear communication with key hospital departments to ensure cross-sector integration and effective implementation of these protocols. Workflows across services were aligned to create a coordinated and responsive patient care process. Key departments supported this integration by adjusting workflows to match the hospital's available resources, helping ensure the continuous and sustainable implementation of the care pathways.

The implementation phase began with small-scale testing through PDSA cycles. These iterative tests allowed teams to assess the functionality and feasibility of each workflow in real-world settings. The project team conducted sector-specific training sessions to build local capability, enabling frontline teams to identify areas requiring refinement and propose context-sensitive improvements. Local leaders and multidisciplinary staff collaboratively refined the workflows to ensure ownership and sustainability of the proposed changes. Once the processes consistently met clinical goals and demonstrated reliable performance, comprehensive training was extended to all hospital personnel. This included both technical instruction and educational initiatives to raise awareness, foster engagement, and deepen understanding of TMAs and their coordinated management. Supplementary Figure S1 summarizes the iterative PDSA process used to plan a pathway change, test it on a small scale, examine performance and frontline feedback, and adopt, adapt, or abandon the change before the next cycle.

### Electronic medical records support

To enhance clinical decision-making and streamline case detection, the Health Information Technology department at EHI developed tailored tools within the electronic medical record (EMR) system. These tools incorporated decision trees aligned with the institutional care pathways and were integrated across key hospital departments. Specific laboratory results automatically triggered alerts to the clinical team, prompting timely recognition of potential TMA cases. These alerts facilitate early diagnostic suspicion, accelerate the ordering of confirmatory tests, and support a structured, protocol-driven diagnostic and therapeutic approach.

ADAMTS13 activity was measured with the fully automated *HemosIL AcuStar* ADAMTS13 Activity chemiluminescent immunoassay (Werfen, Spain) on the ACL AcuStar platform. Inhibitory anti-ADAMTS13 antibodies were assessed using a Bethesda-based mixing method with the same activity assay (20).

Soluble C5b-9 testing was not initially available for physicians to order. After laboratory implementation, it was added to the institutional test catalog and incorporated into the EMR *CareSet* for patients with suspected post-transplant TMA (18, 21). Its results are interpreted together with clinical and laboratory criteria, rather than as a standalone diagnostic requirement.

### Indicators and monitoring

To monitor the Center of Excellence's performance and assess improvements over time, a structured set of performance indicators was established. These included outcome, process, and balancing measures:

- Outcome indicator: Average time from initial clinical suspicion to diagnostic confirmation and initiation of treatment.
- Process indicator: Proportion of patients managed according to the established TMA care pathway.
- Balancing indicator: Average length of stay of TMA patients in the ICU (used to monitor unintended consequences and system pressures).

Cross-cutting measures will be complemented by disease-specific outcomes, which will be collected and analyzed prospectively. For TTP, these include time to platelet response, time to recovery of ADAMTS13 activity, exacerbation, and clinical or biochemical relapse during follow-up. For complement-mediated TMA, measures include renal recovery, need for kidney replacement therapy, and dialysis duration. For TA-TMA, measures include hematologic and organ responses, non-relapse mortality, and overall survival.

After implementation, ongoing monitoring was assigned to the Medical Practice Department, which is responsible for tracking the whole patient journey through the institution's care appropriateness program. This unit will oversee the defined indicators and coordinate updates to workflows and protocols as necessary.

## Practical points

Supplementary Figure S2 shows the integrated diagnostic workflow developed for early detection and management of TMA in the EHI setting. The process starts with identifying suspected cases—either through clinical evaluation, laboratory or imaging findings, or previous diagnoses. A detailed assessment by the clinical team determines whether the suspicion is confirmed or ruled out. In confirmed cases, a structured protocol is initiated within the EMR, which involves completing the PLASMIC score (platelet count, hemolysis, no active cancer, no history of solid-organ or stem-cell transplant, mean corpuscular volume, international normalized ratio, and creatinine), ordering a predefined diagnostic panel, and activating bed management and clinical support teams (hematology and nephrology). The diagnostic approach is customized for specific patient groups (adults, children, pregnant women, post-renal transplant, and post-hematopoietic stem cell transplant), with automated decision support guiding the next steps.

Figures 1A,B detail the specific therapeutic pathways for suspected TTP and HUS, respectively. Figure 1A presents the TTP management flow, which includes pre-test probability assessment using PLASMIC or French scores and determination of ADAMTS13 activity. Treatment decisions, such as initiating plasmapheresis, prescribing corticosteroids, and considering rituximab, are based on stratified risk levels.

**Figure 1:**
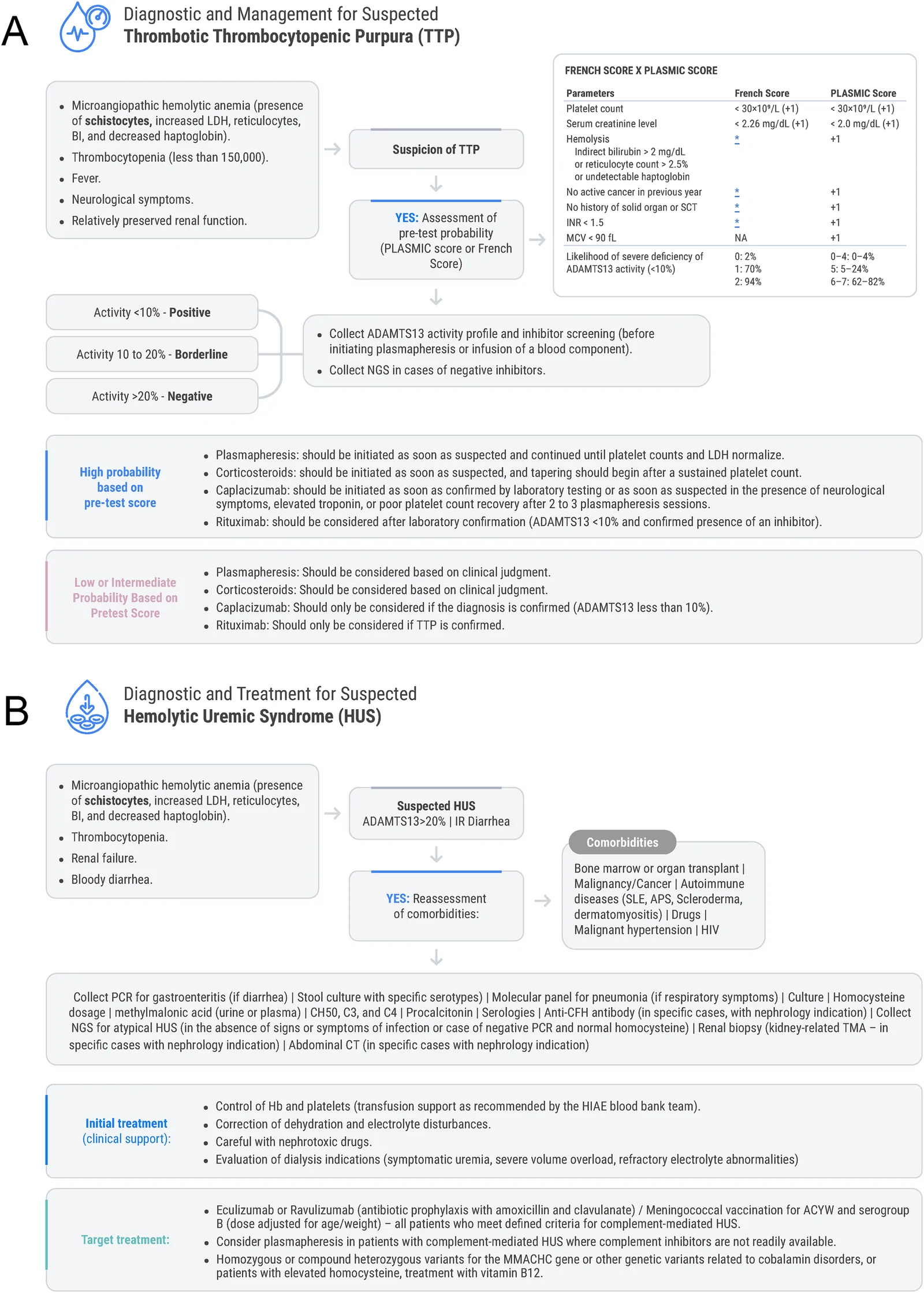
An integrated workflow was developed for the early detection and management of thrombotic thrombocytopenic purpura **(panel A)** and hemolytic uremic syndrome **(panel B)**. ADAMTS13, a disintegrin and metalloproteinase with thrombospondin type 1 motif, member 13; APS, antiphospholipid syndrome; BI, indirect bilirubin; C3, complement component 3; C4, complement component 4; CFH, complement factor H; CH50, total hemolytic complement activity; CT, computed tomography; Hb, hemoglobin; HIV, human immunodeficiency virus; HUS, hemolytic uremic syndrome; LDH, lactate dehydrogenase; MCV, mean corpuscular volume; MMACHC, methylmalonic aciduria and homocystinuria type C gene; NA, not applicable; NGS, next-generation sequencing; PCR, polymerase chain reaction; SCT, stem cell transplantation; SLE, systemic lupus erythematosus; TMA, thrombotic microangiopathy; TTP, thrombotic thrombocytopenic purpura.

Figure 1B outlines therapeutic pathways for suspected HUS. The algorithm emphasizes differential diagnosis based on ADAMTS13 activity, history of diarrhea, and infectious or autoimmune causes. Supportive care is prioritized early on, with targeted treatments, such as eculizumab, guided by the underlying cause of HUS and supported by additional investigations, including genetic testing.

Figure 2A illustrates the integration of clinical decision support within the EMR system to optimize the early detection and management of TMA. When predefined laboratory criteria are met—such as the presence of schistocytes >1% and thrombocytopenia—an automated alert is triggered within the EMR. This alert notifies the clinical team and prompts further investigation, including the ordering of ADAMTS13 activity testing and inhibitor screening. The system also provides access to a structured decision-support interface that includes the PLASMIC score calculator, risk-based treatment recommendations, and a prescription menu tailored by patient profile (e.g., diagnosis code, age group, pregnancy status). These functionalities were developed to ensure timely recognition, guide evidence-based decisions, and standardize care pathways across departments.

**Figure 2:**
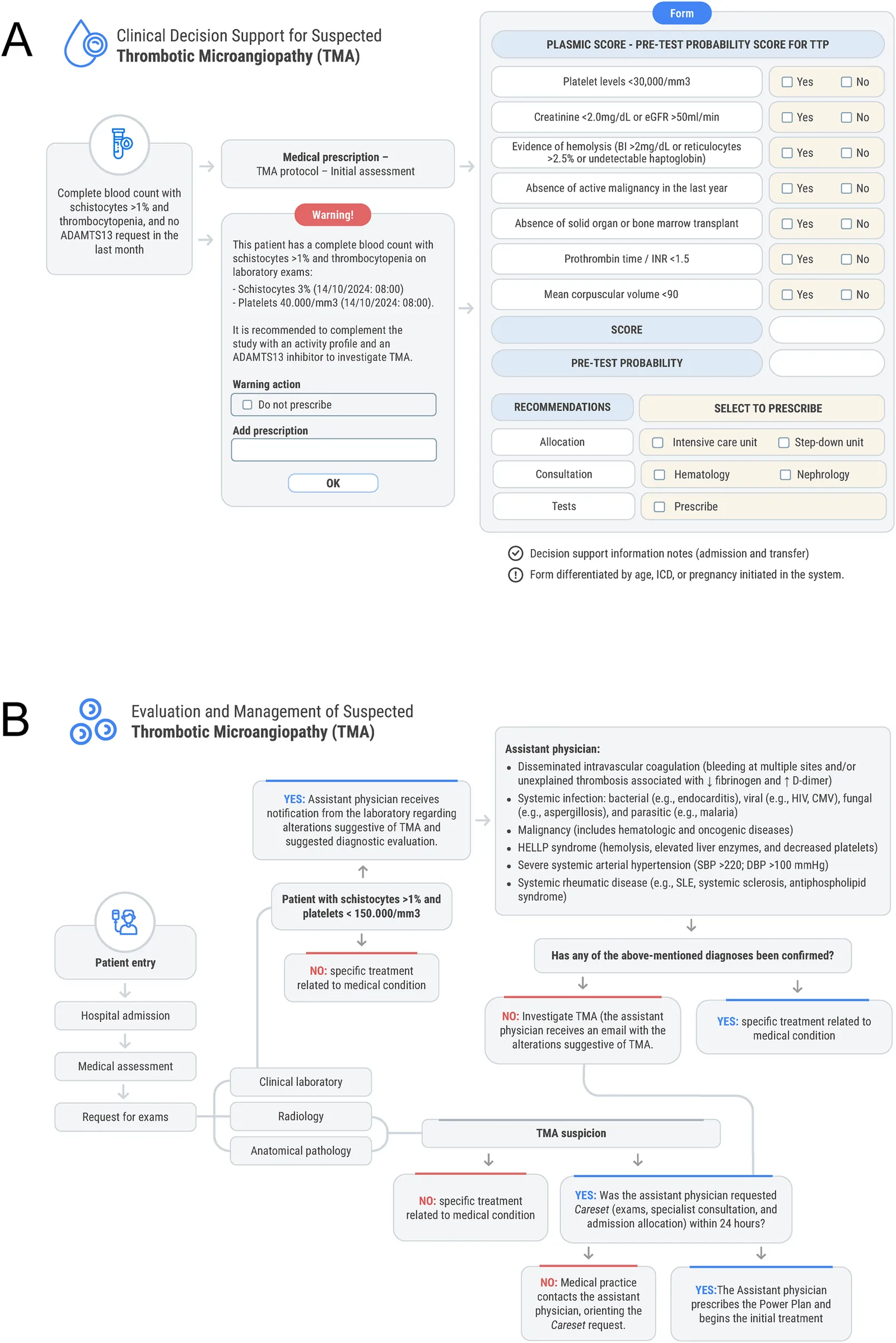
Panel **(A)**—decision-making tool to early detect thrombotic microangiopathies integrated into the electronic medical records. ADAMTS13, a disintegrin and metalloproteinase with thrombospondin type 1 motif, member 13; BI, indirect bilirubin; eGFR, estimated glomerular filtration rate; ICD, International Classification of Diseases; INR, international normalized ratio; TMA, thrombotic microangiopathy; TTP, thrombotic thrombocytopenic purpura. Panel **(B)**—Monitoring workflow aligned with medical practice at the *Einstein Hospital Israelita*, Brazil. CMV, cytomegalovirus; DBP, diastolic blood pressure; HELLP, hemolysis, elevated liver enzymes, and low platelet count; HIV, human immunodeficiency virus; SBP, systolic blood pressure; SLE, systemic lupus erythematosus; TMA, thrombotic microangiopathy.

Finally, Figure 2B presents the structured monitoring flow for patients with suspected or confirmed TMA. The process begins at hospital admission, with medical evaluation and initial diagnostic testing, including laboratory, radiology, and pathology assessments. If schistocytes >1% and thrombocytopenia (<150,000) are identified, clinical suspicion of TMA is raised. A decision-support checklist guides clinicians in evaluating alternative diagnoses, mimics, triggers, and associated conditions, including disseminated intravascular coagulation, sepsis, malignancy, and autoimmune disease, without assuming that the presence of any of these excludes TMA. When TMA is associated with an autoimmune or other primary disorder, management is directed primarily at the underlying disease, while supportive care and phenotype-specific TMA therapy are considered based on clinical severity. A notification system is triggered, prompting the Medical Practice team to coordinate activation of the *CareSet* and Power Plan protocols. This ensures timely prescription, initiation of treatment, and allocation of required resources within 24 h. The entire process is designed to ensure rapid and systematic identification, escalation, and monitoring of TMA cases while maintaining alignment with institutional best practices and patient safety protocols.

## Remarks on implementation

This QI initiative successfully established a multidisciplinary, rapid-response service for TMA within a Brazilian quaternary hospital, integrating evidence-based clinical pathways, EMR decision-support tools, and cross-sector collaboration. The project established structured diagnostic and treatment algorithms tailored to distinct patient populations, including adults, children, pregnant women, and transplant recipients. Key innovations included automated alerts for early TMA detection, embedded scoring systems such as the PLASMIC score (22), and streamlined workflows to reduce time-to-diagnosis and optimize resource allocation. Collectively, these interventions aim to promote early recognition of TMAs, standardize clinical decision-making, and improve coordination across services, thereby supporting sustained improvements in TMA care delivery.

### Operational impact

The Center of Excellence's integrated approach has the potential to significantly improve operational efficiency. By coordinating multidisciplinary evaluation and establishing clear diagnostic pathways, the model reduces fragmentation in care delivery and supports more efficient use of hospital resources. Earlier identification of suspected TMA cases facilitates timely specialist involvement and appropriate allocation of therapeutic resources, ultimately improving care efficiency and supporting better clinical outcomes (7, 23). In addition, standardized workflows and EMR-integrated tools enhance care efficiency by reducing diagnostic uncertainty and minimizing redundant testing or inappropriate interventions (24, 25).

As a result, the EHI Center of Excellence aligns with the IHI’s quintuple aim (26):

- Enhance patient experience through coordinated, transparent, and quicker care with clear escalation pathways, earlier diagnoses, and shared decision-making touchpoints;
- Improve population health by standardizing pathways across sites and implementing longitudinal surveillance, such as ADAMTS13 monitoring and relapse tracking, to decrease recurrent disease;
- Reduce per-capita costs by minimizing avoidable ICU days, shortening hospital stays, limiting the need for renal replacement therapy, and tailoring high-cost therapies using biology-driven triage;
- Support care-team well-being through role clarity, predictable workflows, and fewer after-hours “fire drills,” which could help lessen burnout and cognitive load; and
- Promote health equity by minimizing unwarranted variation with protocolized care and ensuring consistent access regardless of site or patient background.

Collectively, these features position the Center of Excellence as a model for delivering high-value care in complex, high-risk conditions such as TMA (7, 8, 27). Similar approaches have demonstrated meaningful benefits in other settings. For example, in Spain, Rivera *et al*. (23) reported that implementing a hospital-wide multidisciplinary team for TMA was associated with shorter time to treatment, reduced ICU and total hospital length of stay, and a decline in the need for chronic renal replacement therapy in the post-implementation period. The authors attributed these improvements to faster recognition of suspected cases, protocolized clinical responses, and real-time communication among team members.

### TTP-specific clinical pathways and relapse prevention

Merrill *et al*. (2025) (28) used the Define–Measure–Analyze–Improve–Control QI framework to identify failures in usual care, create a multidisciplinary TMA team, standardize acute treatment and discharge coordination, and institute outpatient ADAMTS13 surveillance at intervals of no more than three months. Their report illustrates pathway standardization as a QI intervention. These authors reported an approximately 91% relative risk reduction in 90-day relapse or TTP-related death after establishing a formal TTP clinical pathway supported by a multidisciplinary TMA team (28). The pathway standardized therapeutic plasma exchange (TPE), expanded the use of Rituximab and Caplacizumab, improved coordination among clinical services, and incorporated outpatient biochemical surveillance with ADAMTS13 monitoring—highlighting the value of integrated inpatient–outpatient care models. Our TTP flow (pre-test probability, early ADAMTS13 testing, and risk-stratified therapy) and our indicator set (time-to-diagnosis, pathway adherence, and ICU utilization) were designed with similar objectives: to reduce diagnostic delays, standardize treatment, and maintain vigilance after discharge in accordance with evidence-based recommendations (29). Although this report focuses on the design and feasibility of the program, the established indicators and monitoring plan allow the prospective evaluation of relapse, treatment timeliness, and resource utilization as the program matures.

### Role of ADAMTS13 in pathway triage

In 2015, Bendapudi *et al*. (30) showed that patients with severe ADAMTS13 deficiency (≤10%) formed a biologically and clinically distinct TMA cohort with better survival during TPE and faster platelet recovery than patients without severe deficiency. Although the authors described the severely deficient group as acquired or autoimmune TTP, an inhibitor was not demonstrable in every patient. Severe ADAMTS13 deficiency therefore supports TTP but does not alone prove an immune mechanism; congenital TTP remains a differential diagnosis when inhibitor or anti-ADAMTS13 antibody testing does not establish autoimmunity.20 This evidence informed the pathway branch points embedded in the EMR and supports timely phenotype-directed treatment while limiting unnecessary TPE in non-TTP TMAs.

## Future directions and recommendations

Despite its promising outcomes, implementing a multidisciplinary TMA response model presents several challenges. The initial financial investment required for EMR customization, integration of clinical decision-support tools, and staff training was substantial. Additionally, aligning workflows across different specialties required ongoing coordination and strong leadership to overcome organizational silos and encourage collaboration. Sustaining the initiative also required continuous monitoring and institutional support to maintain the new diagnostic pathways, multidisciplinary coordination, and clinical workflows over time.

Using iterative PDSA cycles was essential in this process. It allowed the team to test, adapt, and enhance interventions in real-world clinical environments. This adaptable approach was crucial for balancing evidence-based recommendations with the operational realities and resource limitations of a large, complex hospital in a resource-limited setting.

Although developed in a large quaternary hospital, several components of this model are transferable to other healthcare settings. The use of standardized diagnostic pathways, multidisciplinary coordination, and predefined laboratory triggers can be implemented even in institutions with more limited resources. In contrast, some elements—such as EMR-integrated decision-support tools and automated alert systems—require more advanced digital infrastructure and institutional investment. These distinctions are important when adapting the model to other hospitals, particularly in middle-income countries where resources and technological capabilities may vary.

## Data Availability

The data analyzed in this study is subject to the following licenses/restrictions: available from the corresponding author upon reasonable request. Requests to access these datasets should be directed to JCCG, joao.guerra@einstein.br.

## Supplementary material

The Supplementary Material for this article can be found online at: https://www.frontiersin.org/articles/10.3389/fmed.2026.1944769/full#supplementary-material

Supplementary Figure 1. Application of iterative Plan–Do–Study–Act cycles to the development and refinement of the institutional TMA pathways. PDSA, Plan–Do–Study–Act; TMA, thrombotic microangiopathy.

Supplementary Figure 2. Integrated diagnostic workflow developed for early detection and management of thrombotic microangiopathies.

## References

[1] George JN. How I treat patients with thrombotic thrombocytopenic purpura-hemolytic uremic syndrome. Blood. (2000) 96:1223–9. 10.1182/blood.V96.4.1223 10942361

[2] Aigner C Schmidt A Gaggl M Sunder-Plassmann G. An updated classification of thrombotic microangiopathies and treatment of complement gene variant-mediated thrombotic microangiopathy. Clin Kidney J. (2019) 12:333–7. 10.1093/ckj/sfz040 31198225 PMC6543965

[3] Brocklebank V Wood KM Kavanagh D. Thrombotic microangiopathy and the kidney. Clin J Am Soc Nephrol CJASN. (2018) 13:300–17. 10.2215/CJN.00620117 29042465 PMC5967417

[4] Abou-Ismail MY Kapoor S Sridhar CD Nayak L Ahuja S. Thrombotic microangiopathies: an illustrated review. Res Pract Thromb Haemost. (2022) 6:e12708. 10.1002/rth2.12708 35615754 PMC9027164

[5] Gordon CE Chitalia VC Sloan JM Salant DJ Coleman DL Quillen K. Thrombotic microangiopathy: a multidisciplinary team approach. Am J Kidney Dis Off J Natl Kidney Found. (2017) 70:715–21. 10.1053/j.ajkd.2017.05.017 28720207

[6] Hanna RM Henriksen K Kalantar-Zadeh K Ferrey A Burwick R Jhaveri KD. Thrombotic microangiopathy syndromes-common ground and distinct frontiers. Adv Chronic Kidney Dis. (2022) 29:149–160.e1. 10.1053/j.ackd.2021.11.006 35817522

[7] Go RS Winters JL Leung N Murray DL Willrich MA Abraham RS. Thrombotic microangiopathy care pathway: a consensus statement for the mayo clinic complement alternative pathway-thrombotic microangiopathy (CAP-TMA) disease-oriented group. Mayo Clin Proc. (2016) 91:1189–211. 10.1016/j.mayocp.2016.05.015 27497856

[8] Thompson GL Kavanagh D. Diagnosis and treatment of thrombotic microangiopathy. Int J Lab Hematol. (2022) 44(Suppl 1:):101–13. 10.1111/ijlh.13954 36074708 PMC9544907

[9] Fox LC Cohney SJ Kausman JY Shortt J Hughes PD Wood EM. Consensus opinion on diagnosis and management of thrombotic microangiopathy in Australia and New Zealand. Intern Med J. (2018) 48:624–36. 10.1111/imj.13804 29582550

[10] McFarlane PA Bitzan M Broome C Baran D Garland J Girard L-P. Making the correct diagnosis in thrombotic microangiopathy: a narrative review. Can J Kidney Health Dis. (2021) 8:20543581211008707. 10.1177/20543581211008707 33996107 PMC8072824

[11] Leisring J Brodsky SV Parikh SV. Clinical evaluation and management of thrombotic microangiopathy. Arthritis Rheumatol. (2024) 76:153–65. 10.1002/art.42681 37610060

[12] Institute for Healthcare Improvement. Model for Improvement. Available online at: https://www.ihi.org/library/model-for-improvement (Accessed March 4, 2026)

[13] Zheng XL Vesely SK Cataland SR Coppo P Geldziler B Iorio A. ISTH Guidelines for the diagnosis of thrombotic thrombocytopenic purpura. J Thromb Haemost. (2020) 18:2486–95. 10.1111/jth.15006 32914582 PMC8146131

[14] Zheng XL Vesely SK Cataland SR Coppo P Geldziler B Iorio A. ISTH Guidelines for treatment of thrombotic thrombocytopenic purpura. J Thromb Haemost. (2020) 18:2496–502. 10.1111/jth.15010 32914526 PMC8091490

[15] Zheng XL Al-Housni Z Cataland SR Coppo P Geldziler B Germini F. 2025 Focused update of the 2020 ISTH guidelines for management of thrombotic thrombocytopenic purpura. J Thromb Haemost. (2025) 23:3711–32. 10.1016/j.jtha.2025.06.002 40533296 PMC13470878

[16] Scully M Rayment R Clark A Westwood JP Cranfield T Gooding R. A British society for haematology guideline: diagnosis and management of thrombotic thrombocytopenic purpura and thrombotic microangiopathies. Br J Haematol. (2023) 203:546–63. 10.1111/bjh.19026 37586700

[17] Evangelidis P Gavriilaki E Tsakiris DA. Thrombotic complications after hematopoietic stem cell transplantation and other cellular therapies. Thromb Update. (2024) 16:100186. 10.1016/j.tru.2024.100186

[18] Schoettler ML Carreras E Cho B Dandoy CE Ho VT Jodele S. Harmonizing definitions for diagnostic criteria and prognostic assessment of transplantation-associated thrombotic microangiopathy: a report on behalf of the European society for blood and marrow transplantation, American society for transplantation and cellular therapy, Asia-Pacific blood and marrow transplantation group, and center for international blood and marrow transplant research. Transplant Cell Ther. (2023) 29:151–63. 10.1016/j.jtct.2022.11.015 36442770 PMC10119629

[19] Agência Nacional de Vigilância Sanitária—ANVISA. Cablivi® (caplacizumabe): novo registro. Available online at: https://www.gov.br/anvisa/pt-br/assuntos/medicamentos/novos-medicamentos-e-indicacoes/cablivi-r-caplacizumabe-novo-registro (Accessed August 28, 2026)

[20] Montaruli B Novelli C Solfietti L Valpreda A Bazzan M Beverina I. Inhibitory anti ADAMTS13 antibodies with a new rapid fully automated CLIA assay. Int J Lab Hematol. (2021) 43:298–304. 10.1111/ijlh.13372 33095979

[21] Schoettler ML Gavriilaki E Carreras E Bo-Kyoung C Dandoy CE Ho VT. An ASTCT, CIBMTR, EBMT, and APBMT consensus statement defining response criteria for hematopoietic cell transplantation associated thrombotic microangiopathy (TA-TMA) directed therapy. Transplant Cell Ther. (2025) 31:610–23. 10.1016/j.jtct.2025.05.028 40514012 PMC12718118

[22] Vyas A Isaac S Kaur D Yadav U. Role of the PLASMIC score in the management of thrombotic thrombocytopenic Purpura. Cureus. (2023) 15:e36188. 10.7759/cureus.36188 37065284 PMC10104422

[23] Uriol Rivera MG Cabello Pelegrin S Ballester Ruiz C López Andrade B Lumbreras J Obrador Mulet A. Impact of a multidisciplinary team for the management of thrombotic microangiopathy. PLoS One. (2018) 13:e0206558. 10.1371/journal.pone.0206558 30388144 PMC6214549

[24] Gallo T Curry SC Heise CW Antonescu CC Raschke RA. Clinical decision support to reduce unnecessary diagnostic testing for heparin-induced thrombocytopenia. Br J Haematol. (2023) 202:1011–7. 10.1111/bjh.18902 37271143

[25] Delvaux N Piessens V Burghgraeve TD Mamouris P Vaes B Stichele RV. Clinical decision support improves the appropriateness of laboratory test ordering in primary care without increasing diagnostic error: the ELMO cluster randomized trial. Implement Sci iS. (2020) 15:100. 10.1186/s13012-020-01059-y 33148311 PMC7640389

[26] Nundy S Cooper LA Mate KS. The quintuple aim for health care improvement: a new imperative to advance health equity. JAMA. (2022) 327:521–2. 10.1001/jama.2021.25181 35061006

[27] Germeni E Cooper J Briggs A Laurence J. Treatment discontinuation in adults with atypical hemolytic uremic syndrome (aHUS): a qualitative study of international experts’ perspectives with associated cost-consequence analysis. BMC Nephrol. (2024) 25:411. 10.1186/s12882-024-03770-0 39543505 PMC11566521

[28] Merrill SA Yu S Webber SE Gotses J Platt EL Arays R. The TMA team and TTP pathway improved outcomes in a cohort with thrombotic thrombocytopenic purpura. PLoS One. (2025) 20:e0325417. 10.1371/journal.pone.0325417 40478853 PMC12143514

[29] Connelly-Smith L Alquist CR Aqui NA Hofmann JC Klingel R Onwuemene OA. Guidelines on the use of therapeutic apheresis in clinical practice—evidence-based approach from the writing committee of the American society for apheresis: the ninth special issue. J Clin Apheresis. (2023) 38:77–278. 10.1002/jca.22043 37017433

[30] Bendapudi PK Li A Hamdan A Uhl L Kaufman R Stowell C. Impact of severe ADAMTS13 deficiency on clinical presentation and outcomes in patients with thrombotic microangiopathies: the experience of the Harvard TMA research collaborative. Br J Haematol. (2015) 171:836–44. 10.1111/bjh.13658 26314936

